# Aquaporin ZmTIP2;3 Promotes Drought Resistance of Maize through Symbiosis with Arbuscular Mycorrhizal Fungi

**DOI:** 10.3390/ijms25084205

**Published:** 2024-04-10

**Authors:** Deyin Wang, Ying Ni, Kailing Xie, Yuanhao Li, Wenxiang Wu, Hanchen Shan, Beijiu Cheng, Xiaoyu Li

**Affiliations:** Key Laboratory of Crop Stress Resistance and High Quality Biology of Anhui Province, Anhui Agricultural University, Hefei 230036, China; 15754366256@139.com (D.W.); 15215517537@163.com (Y.N.); 15056907002@163.com (K.X.); liyuanhao04@163.com (Y.L.); 15255159313@163.com (W.W.); shanhc2022@163.com (H.S.)

**Keywords:** maize, aquaporin, arbuscular mycorrhizal, drought, symbiosis

## Abstract

Arbuscular mycorrhizal fungi symbiosis plays important roles in enhancing plant tolerance to biotic and abiotic stresses. Aquaporins have also been linked to improved drought tolerance in plants and the regulation of water transport. However, the mechanisms that underlie this association remain to be further explored. In this study, we found that arbuscular mycorrhiza fungi symbiosis could induce the gene expression of the aquaporin ZmTIP2;3 in maize roots. Moreover, compared with the wild-type plants, the maize *zmtip2;3* mutant also showed a lower total biomass, colonization rate, relative water content, and POD and SOD activities after arbuscular mycorrhiza fungi symbiosis under drought stress. qRT-PCR assays revealed reduced expression levels of stress genes including *LEA3*, *P5CS4*, and *NECD1* in the maize *zmtip2;3* mutant. Taken together, these data suggest that *ZmTIP2;3* plays an important role in promoting maize tolerance to drought stress during arbuscular mycorrhiza fungi symbiosis.

## 1. Introduction

Drought is a worldwide environmental issue, and because of global climate change, drought is becoming worse in (semi-) arid regions [1,2]. Maize yield is influenced by a range of factors, including a myriad of biotic and abiotic stresses, with drought being a primary constraint in agricultural production. Drought can trigger changes in crop morphology, physiological conditions, and metabolic pathways, thereby impacting crop productivity [1]. Severe drought can result in a substantial decrease in maize yield [3]. Hence, exploring maize’s stress response to drought conditions and unraveling its protective mechanisms are of the utmost importance for maintaining maize production and supporting economic growth.

Aquaporins (AQPs), as a multifunctional protein family of the major intrinsic protein (MIP) superfamily, are ubiquitous in almost all organisms [4]. AQPs, which are the primary water transport channels in the plasma membrane and the majority of plant cell intracellular compartments, contribute to the osmotic regulation of organisms [5,6]. Certain AQP genes are upregulated during dehydration to support water transport and preserve regular physiological functions, while the expression of other genes is downregulated to lower total water permeability and prevent excessive water loss [7]. A variety of cellular and developmental processes are also impacted by AQPs [8]. While *AtPIP1;4* and *AtPIP2;5* are upregulated, PIP transcripts in *Arabidopsis* leaves generally show a gradual downregulation under drought stress [9]. The constitutive expression of *AtPIP2;6* is not significantly affected by drought stress [9]. Under drought stress, the accumulation of *NtPIP1;1* and *NtPIP2;1* transcripts significantly decrease, while only the accumulation of *NtAQP1* transcripts increase [10]. *ScPIP1*-overexpressing *Arabidopsis* plants have exhibited higher germination rates, longer roots, and higher survival rates compared to wild-type plants under drought and salt stresses [11]. Maize plants overexpressing *ZmPIP2;5* have shown increased leaf water potential and hydraulic conductivity of their roots under hyperosmotic conditions, enhancing the plant’s ability to withstand abiotic stresses [12].

Furthermore, plants can also benefit from symbiosis with arbuscular mycorrhizal fungi (AMF) to survive drought stress. This symbiotic relationship between AMF and the root system of higher plants is prevalent in most terrestrial ecosystems. It is estimated that about 90% of terrestrial plant species can form mutualistic relationships with arbuscular mycorrhiza fungi (AMF), and more than 80% of vascular plants (including major crops) can form mycorrhizal symbionts with AMF [13]. On the one hand, this symbiotic relationship is manifested by the massive extension of mycelium from outside the roots to the surrounding rhizosphere after colonization by AM fungi, thus forming a network of mycelium that facilitates connections between the roots and the soil moisture and assists in the uptake of water and nutrients that the host plant finds difficult to absorb during drought conditions, and on the other hand, through these mycelium, minerals such as phosphate, ammonium, nitrate, sulphate, and potassium are absorbed from the soil and supplied to the plant in exchange for carbohydrates and fats, thereby sustaining the growth and metabolism of the plant itself and completing its life cycle [14,15,16,17]. Maurel and Plassard [18] recognized the significance of aquaporins in the mycorrhizal symbiosis process for nutrient and water exchange. The findings of Bárzana et al. [19] further supported this viewpoint, revealing that among 36 maize aquaporins, 16 were regulated by AM fungi under drought stress [20]. However, the results regarding AM symbiosis on aquaporin regulation so far indicate that the symbiosis’s impact on aquaporin gene expression is intricate and depends on the intrinsic properties of osmotic stress. Moreover, as demonstrated by Bárzana et al. [19] on maize aquaporins, the regulation of plant aquaporins also depends on the intensity and duration of the applied stress. Considering the diversity of substrates transported by aquaporin isoforms regulated by AM, they may play roles in regulating leaf and root hydraulic conductance and other physiological processes such as nutrient uptake and transport, stomatal movement, carbon fixation [21], or signaling processes [22]. Therefore, elucidating the effects on plant growth and the development of aquaporins regulated by AM in plants is necessary to understand their role in AM-induced drought resistance. 

In this study, by analyzing previous transcriptome data, we identified a candidate AQP gene member, *ZmTIP2;3*, from the family of AQP genes found in maize (*Zea mays* L.), which was significantly upregulated by AM fungi. Our focus was on exploring the symbiotic interaction between AMF and maize aquaporins under drought stress conditions. The results indicate that *ZmTIP2;3* was significantly induced upon drought treatment during AM fungi symbiosis. The biomass, colonization rate, relative water content (RWC), photosynthesis, POD and SOD activities, proline content, and the expression of other drought-related genes, *LEA3*, *P5CS4* and *NECD1*, were lower in the *zmtip2;3* mutant than those in the wild type after inoculation with the AM fungi under drought stress. These results indicated that *ZmTIP2;3* was influential in promoting maize’s tolerance to drought stress during AM fungi symbiosis. This evolutionary mechanism provides us with a multi-dimensional perspective on the various ways in which plants adapt to adverse conditions. Our research enriches the understanding of the intricate adaptations of plants to stress and adds breadth to the pathways for plant resistance against adverse stresses. This provides a theoretical basis for breeding new varieties of maize resistant to drought.

## 2. Results

### 2.1. Bioinformatics Analysis of the ZmTIP2;3 Gene

**Figure 1:**
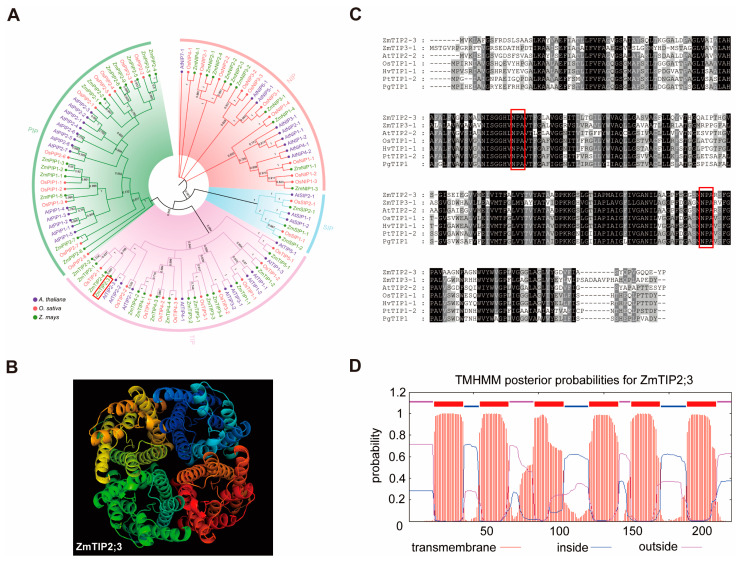
Bioinformatics analysis and characterization of ZmTIP2;3. (**A**) Phylogenetic analysis of amino acid sequences of maize aquaporins with other aquaporins of different species. Red boxes showed ZmTIP2;3; (**B**) predicted three-dimensional structure of the ZmTIP2;3 protein. The different colors represent the four monomers composing the ZmTIP2;3 protein; (**C**) multiple alignments of ZmTIP2;3 (NP_001358543) with other known TIPs. The depth of color represents the degree of similarity between amino acids. The exact sequence was highlighted in black color. Red boxes showed two highly conserved NPA motifs; (**D**) distribution of transmembrane domains in ZmTIP2;3 (red represents the transmembrane, blue represents the inner membrane, and purple represents the outer membrane).

The AQP genes in *Zea Mays* were used to construct a phylogenetic tree compared with those in *Arabidopsis* and *Oryza sativa* (Figure 1A). Similar to the OsAQPs and AtAQPs, the phylogenetic analysis revealed that the ZmAQPs also clustered into four distinct subfamilies (PIPs, TIPs, NIPs, and SIPs). Among the 41 identified ZmAQPs proteins, 13 belonged to PIPs, 15 to TIPs, 10 to NIPs and 3 to SIPs. TIPs are a family of vesicular membrane-intrinsic proteins, and vesicles are important for the maintenance of cell morphology and osmotic pressure homeostasis. Within the TIP2 family, ZmTIP2;3 is closely related to ZmTIP1;1, ZmTIP2;1, and ZmTIP2;2, which have already been reported to possess the ability of water transport and resistance to water stress, suggesting that *ZmTIP2;3* is also related to water transport. Pymol software (version 3.0) was used to visualize the protein’s three-dimensional structure (Figure 1B). The ZmTIP2;3 protein possesses a hydrophobic pore in the center of each monomer for passive solute transfer, giving it the typical tetramer structure of aquaporin family members. To further determine the similarity between ZmTIP2;3 and other reported TIPs related to drought, amino acid sequences of ZmTIP2;3, ZmTIP3;1, AtTIP2;2, OsTIP1;1, HvTIP1;1, PtTIP1;2, and PgTIP1 were compared using ClustalX (version 1.81) and GENEDOC (version 2.7) (Figure 1C). The results showed that ZmTIP2;3 contained two conserved NPA motifs (Asn-Pro-Ala). The TMHMM website was used to predict the potential transmembrane domains from the amino acid sequence of ZmTIP2;3 and the result revealed that it contained six transmembrane domains (Figure 1D).

### 2.2. Subcellular Localization of ZmTIP2;3

**Figure 2:**
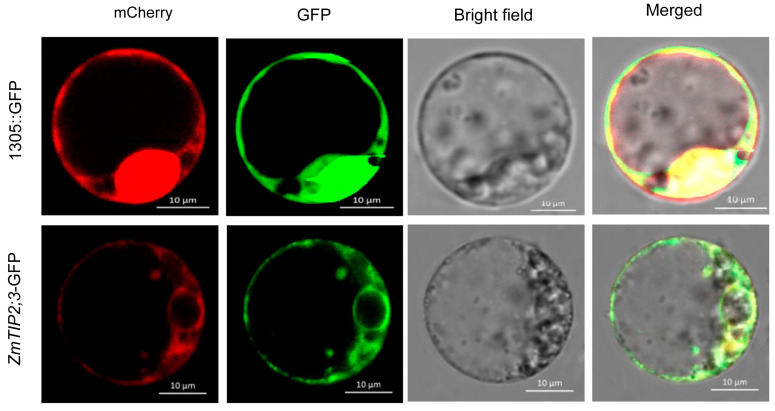
Subcellular localization of ZmTIP2;3. Four visual fields were observed: red channel (mCherry), green channel (GFP), bright channel (brightfield), and merged. Scalebar = 10 µm.

The full length of the *ZmTIP2;3* gene and the length of the coding region are 1355 bp and 747 bp, respectively, which contain three exons and two introns. The amino acid sequence encoded by *ZmTIP2;3* is 248 aa. The coding sequence of *ZmTIP2;3* was cloned from maize roots using cDNA amplification methods. To investigate the subcellular localization of ZmTIP2;3, a *ZmTIP2;3*::GFP fusion construct was generated under the control of the CaMV 35S promoter. The results showed that the *ZmTIP2;3*::GFP fusion protein was found in the cell membrane and the nuclear membrane, whereas the 1305::GFP control was found in both the cell membrane and the nucleus, indicating that ZmTIP2;3 was a membrane-localized protein (Figure 2).

### 2.3. Mycorrhizal-Inducible Expression of the ZmTIP2;3 Gene

**Figure 3:**
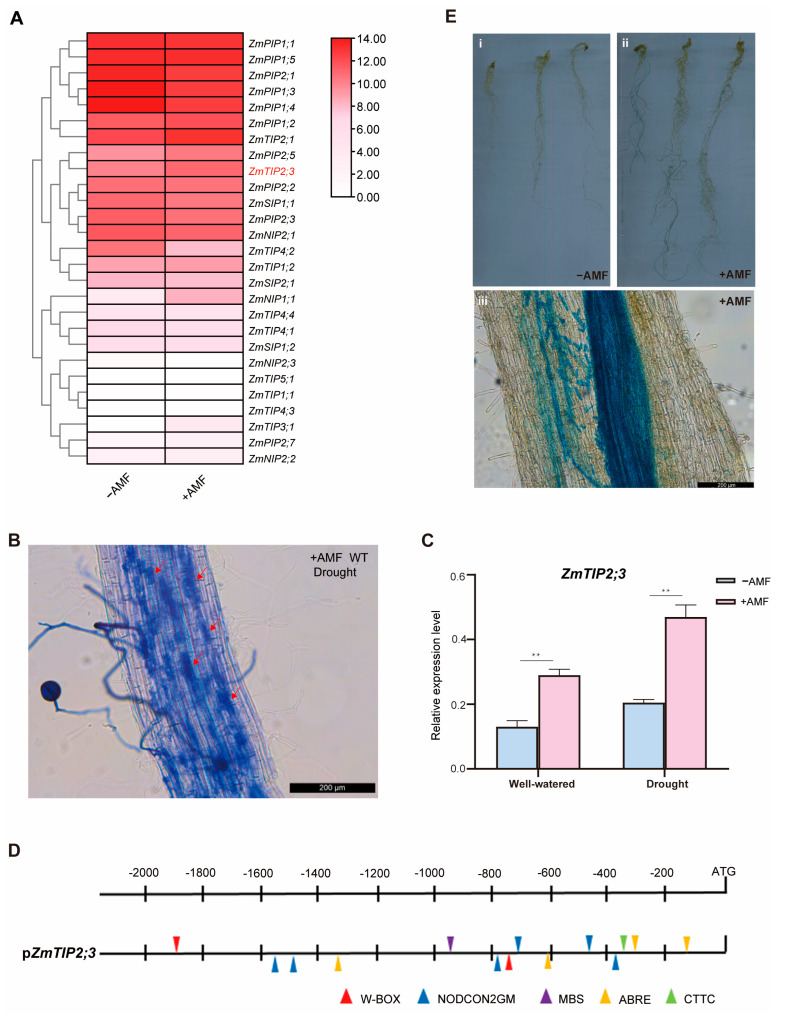
Mycorrhizal-inducible expression of the *ZmTIP2;3* gene. (**A**) Heatmap of the *ZmAQP* family gene expression induced by AMF. (**B**) Trypan blue staining of mycorrhizal roots. The red arrows indicate arbuscule. Scalebar = 200 µm. (**C**) The relative expression level of *ZmTIP2;3* in maize roots inoculated with AMF and non-inoculated controls measured was by qRT-PCR. (**D**) Analysis of cis-acting elements in the promoter of *ZmTIP2;3* gene. (**E**) Analysis and X-Gluc staining of hairy roots of *L. japonicus* expressing p*ZmTIP2;3*::GUS in the absence or presence of AMF. Figure. i shows the hairy roots that are not inoculated with AM fungi and figure. ii shows the hairy roots that are inoculated with AM fungi. figure. iii is a larger version of figure ii. Scalebar = 200 µm. Standard deviation was obtained from three independent replicates. An asterisk indicates significant differences determined by Student’s *t* test, ** *p* ≤ 0.01.

The analysis of the transcriptome data revealed that the expression levels of *ZmTIP2;3* were significantly upregulated with AMF (Figure 3A). To explore the expression levels of *ZmTIP2;3* after inoculation with AM fungi under drought stress, maize plants were grown in a sand/vermiculite/perlite mixture-based substrate inoculated or mock-inoculated with AM fungi and supplemented with sufficient or deficient water. At 8 weeks post inoculation, trypan blue staining of the roots revealed well-developed arbuscule and mycelium structures in the maize roots under drought conditions, indicating that a symbiotic relationship between the maize roots and AM fungi had been formed (Figure 3B). The relative expression level of *ZmTIP2;3* of the mycorrhizal maize roots showed a statistically significant increase compared with the non-mycorrhizal roots whether under sufficient water or drought conditions (Figure 3C), which corresponded to the transcriptome data (Figure 3A), suggesting that the expression level of *ZmTIP2;3* was induced by AMF, especially under drought conditions.

To further confirm that *ZmTIP2;3* was induced by AM fungi and was associated with drought, the online tool RSAT (https://rsat.eead.csic.es/plants/dna-pattern_form.cgi (accessed on 2 January 2024)) was used to analyze the predicted 2000 bp promoter region upstream of *ZmTIP2;3*. The results revealed one cis-acting element, MBS (YAACTG), related to drought stress, six cis-acting elements, NODCON2GM (CTCTT), and one CTTC motif (TCTTGTT) associated with mycorrhizal induction, and two W-BOX (TTGACY) in the promoter region of *ZmTIP2;3* (Figure 3D). Subsequently, a p*ZmTIP2;3*::GUS fusion construct was generated to further verify that the *ZmTIP2;3* was specifically induced by AM fungi. Under AM symbiosis, the hairy roots of *L. japonicus* expressing p*ZmTIP2;3*::GUS were stained blue by X-Gluc dye, suggesting GUS was expressed, while the hairy roots not inoculated with AM fungi were not stained blue (Figure 3E). 

### 2.4. ZmTIP2;3 Promoted Drought Resistance during AM Fungi Symbiosis in Maize

**Figure 4:**
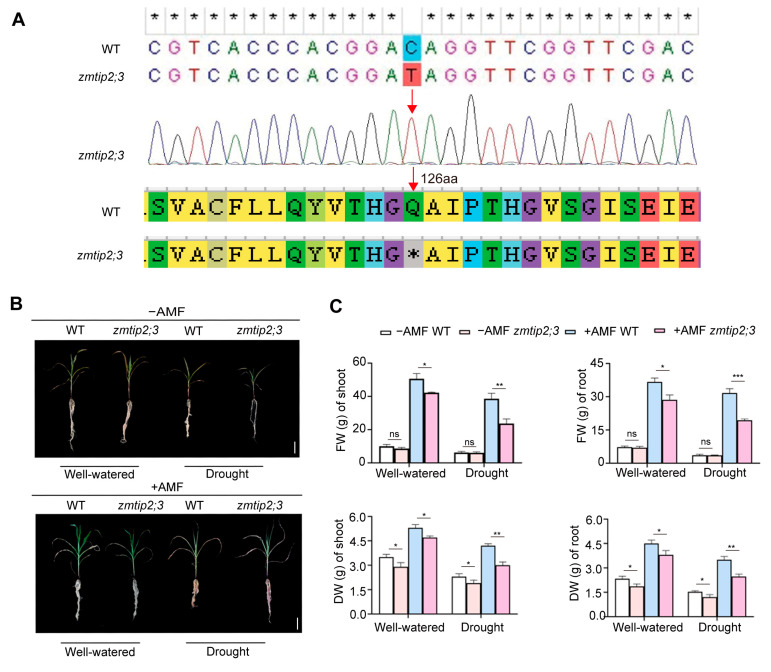
Loss function of ZmTIP2;3 reduced the biomass of maize. (**A**) EMS mutagenesis site in *zmtip2;3* mutant. The red arrows indicate the mutation site. (**B**) Phenotypes of wild-type plants (B73) and *zmtip2;3* mutant plants. Scalebar = 10 cm. (**C**) Fresh and dry weights of wild type and *zmtip2;3* mutant. Standard deviation was obtained from three independent replicates. An asterisk indicates significant differences determined by Student’s *t* test, * *p* ≤ 0.05, ** *p* ≤ 0.01 and *** *p* ≤ 0.001. ns represents no significant difference.

To further verify the function of the *ZmTIP2;3* gene, the *zmtip2;3* maize mutant was obtained. The amino acid sequence changed from glutamine to a stop codon as a result of a single base substitution in the 376 bp base of the coding region sequence initiated by the ATG of the *ZmTIP2;3* (Figure 4A). In order to analyze the effects of AM fungi on the drought resistance of *ZmTIP2;3*, the maize EMS mutant *zmtip2;3* was used to study the phenotypic differences between symbiotic and non-symbiotic maize under drought stress. This study evaluated four treatments: the −AMF (not inoculated with AMF) and well-watered treatment, the −AMF and drought treatment, the +AMF (inoculated with AMF) and well-watered treatment, and the +AMF and drought treatment. The B73 and *zmtip2;3* mutant maize plants were grown in a sand/vermiculite/perlite mixture-based substrate inoculated or mock-inoculated with AM fungi and supplemented with sufficient or deficient water, respectively. After a growth duration of 7 weeks, the maize plants were subjected to two different moisture treatments for a duration of 2 weeks: normal moisture and drought. The relevant phenotypes were then statistically analyzed. The results showed that the maize inoculated with AM fungi grew better than the uninoculated maize, and the wild type grew better than the *zmtip2;3* mutant, for example, with brighter green leaves and more developed roots (Figure 4B). The results showed that the maize inoculated with AM fungi grew better than that without AM fungi, and the wild-type maize also grew better than the mutant maize. The maize inoculated with AM fungi had greener leaves and well-developed root systems, while the leaves of the non-symbiotic maize were wilted and fewer in number compared to the symbiotic maize. Under drought stress, both the non-inoculated wild-type and *zmtip2;3* mutant plants exhibited more severe wilting. As shown in Figure 4C, under drought stress, the shoot fresh weight of the symbiotic mutant *zmtip2;3* maize decreased significantly by 39.6% compared to the wild-type maize, and the root fresh weight decreased significantly by 53.0%. In addition, both the shoot and root dry weights were significantly lower in the symbiotic mutant compared to the wild type. Under well-watered conditions, the shoot fresh weight of the symbiotic mutant *zmtip2;3* maize decreased by 16.4% and the root fresh weight decreased by 29.1% compared to the wild-type maize.

**Figure 5:**
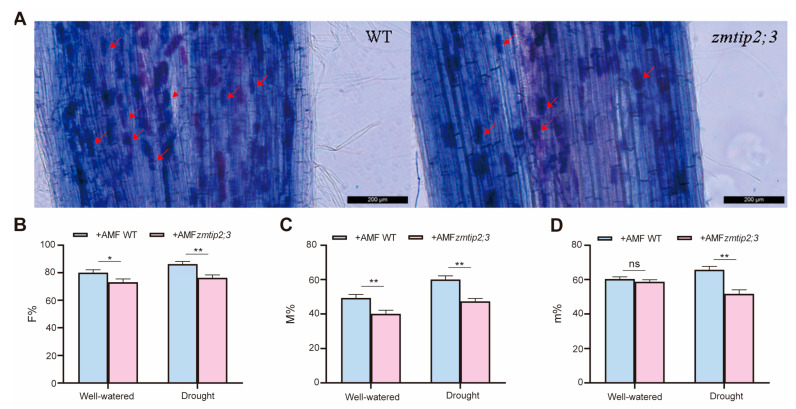
*ZmTIP2;3* promoted mycorrhizal colonization under drought conditions. (**A**) Trypan blue staining of mycorrhizal roots of wild type and *zmtip2;3* mutant at 8 weeks post-inoculation with *G. intraradices*. The scale bar is 50 µm. The red arrows indicate arbuscule. (**B**) F% (frequency of mycorrhiza in the root system) of wild type and *zmtip2;3* mutant. (**C**) M% (intensity of the mycorrhizal colonization in the root system) of wild type and *zmtip2;3* mutant. (**D**) m% (intensity of the mycorrhizal colonization in the root fragments) of wild type and *zmtip2;3* mutant. Standard deviation obtained from three independent replicates. An asterisk indicates significant differences determined by Student’s *t* test, * *p* ≤ 0.05 and ** *p* ≤ 0.01. ns represents no significant difference.

The colonization rate of mycorrhizal fungi directly affects the water and nutrient absorption capacity as well as the stress resistance of plants through the mycorrhizal pathway. In order to investigate the effect of drought conditions on the mycorrhizal colonization rate of the wild type and *zmtip2;3* mutant inoculated with AM fungi at 8 weeks, the roots were stained with trypan blue and the frequency and intensity of infection were measured. The trypan blue staining revealed that both the mutant and wild-type plants had established symbiotic relationships with AM fungi, and normal arbuscular structures were detected in their roots (Figure 5A). We found that the frequency of mycorrhiza in the root system (F%) of *zmtip2;3* was lower than that of the wild type. Additionally, the decline rate during drought stress was 11.6% higher in the wild type compared to the *zmtip2;3* mutant under well-watered conditions (Figure 5B). Under both well-watered and drought stress conditions, the intensity of the mycorrhizal colonization in the root system (M%) of the *zmtip2;3* mutant was significantly reduced by 18.9% and 21.1% compared to the wild type (Figure 5C). Furthermore, the expression levels of both the *zmtip2;3* mutant and the wild type were higher under drought stress compared to well-watered conditions. Under drought stress, the intensity of the mycorrhizal colonization in the root fragments (m%) of the *zmtip2;3* mutant was significantly reduced by 21.3% compared to the wild type, while under well-watered conditions, the m% of the *zmtip2;3* mutant was reduced by 2.8% compared to the wild type (Figure 5D).

**Figure 6:**
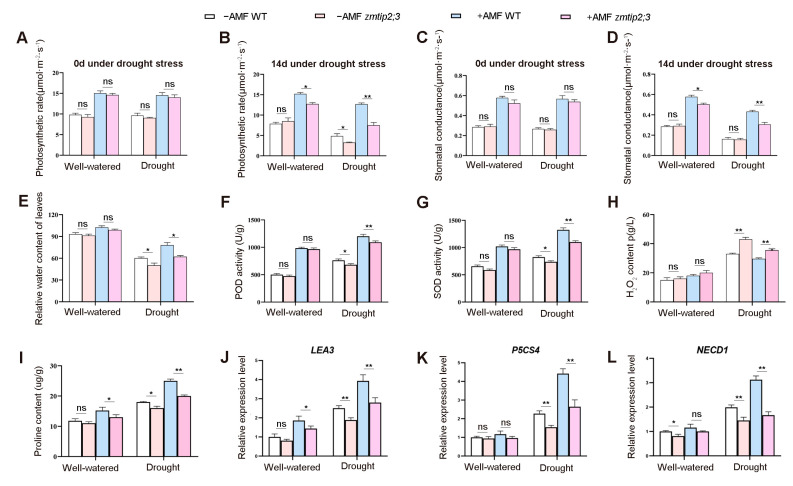
Loss function of ZmTIP2;3 reduced maize drought resistance. (**A**) Photosynthetic rate of 0 d under drought stress. (**B**) Photosynthetic rate of 14 d under drought stress. (**C**) Stomatal conductance of 0 d under drought stress. (**D**) Stomatal conductance of 14 d under drought stress. (**E**) Relative water content of leaves. (**F**) POD activity of leaves. (**G**) SOD activity of leaves. (**H**) H_2_O_2_ content of leaves. (**I**) Proline content of leaves. (**J**) Relative expression level of *LEA3*. (**K**) Relative expression level of *P5CS4*. (**L**) Relative expression level of *NECD1*. Standard deviation obtained from three independent replicates. An asterisk indicates significant differences determined by Student’s *t* test, * *p* ≤ 0.05 and ** *p* ≤ 0.01. ns represents no significant difference.

To evaluate the photosynthetic characteristics of the *zmtip2;3* mutant under drought stress, the photosynthetic rate and stomatal conductance of the wild-type and *zmtip2;3* mutant maize leaves after 7 and 9 weeks of symbiosis with AM fungi were measured using a photosynthesis analyzer. The results showed that at the beginning of drought stress, there were no significant differences in the photosynthetic rate and stomatal conductance between the *zmtip2;3* mutant and wild type under both symbiotic and non-symbiotic conditions (Figure 6A,C). Under normal water conditions, the photosynthetic rate and stomatal conductance in the symbiotic *zmtip2;3* mutant maize were significantly lower compared to the wild type, while there were no significant differences observed between the non-symbiotic *zmtip2;3* mutant maize and the wild type for both traits (Figure 6B,D).

After 14 days of drought stress, the photosynthetic rate and stomatal conductance of the symbiotic *zmtip2;3* mutant maize plants decreased significantly by 58.1% and 41.2%, respectively, compared to the wild type. Under normal water conditions, the symbiotic *zmtip2;3* mutant maize showed a significant decrease of approximately 19.6% in the photosynthetic rate and 14.9% in the stomatal conductance compared to the wild type. Under non-symbiotic conditions, there were no significant differences observed in both traits between the *zmtip2;3* mutant and wild-type maize plants except for the photosynthetic rate. These results indicated that AM fungi symbiosis induced the expression of *ZmTIP2;3* to enhance the photosynthetic capacity of maize under drought stress.

When plants encounter drought stress, a series of physiological and biochemical reactions occur, in which the water status is an important indicator of plant resistance in water-deficient environments. The relative water content intuitively reflects the relationship between the plant water status and transpiration, serving as an important manifestation of a plant’s ability to withstand drought. To investigate the role of the *ZmTIP2;3* gene in enhancing drought resistance by increasing water absorption, the relative water content of plant leaves was studied. Under drought stress, both the symbiotic *zmtip2;3* mutant and wild-type maize had a higher relative water content in their leaves compared to the non-inoculated maize plants (Figure 6E). However, the relative water content of the symbiotic *zmtip2;3* mutant was significantly decreased by 25.8% compared to the wild type, while the non-symbiotic *zmtip2;3* mutant exhibited a decrease of 19.8% compared to the wild type. Under well-watered conditions, there were no significant differences in the relative water content of the symbiotic and non-symbiotic *zmtip2;3* mutants compared to the wild type. These results indicate that the knockout of the *ZmTIP2;3* gene significantly reduced the ability of water absorption and transport under drought stress, leading to the decreased relative water content in the leaves, with a more pronounced effect under symbiotic conditions. This suggested that AM fungi symbiosis induced the expression of the *ZmTIP2;3* gene, enhancing water absorption and transport, and thereby improving the drought resistance of maize.

To further understand the impact of symbiotic and non-symbiotic *zmtip2;3* mutant maize on the antioxidant enzyme activity under drought stress, the activities of peroxidase (POD) and superoxide dismutase (SOD) in the symbiotic maize leaves with AM fungi for 9 weeks were examined. Under normal water conditions, there was no significant difference in the POD activity between the non-inoculated wild-type and *zmtip2;3* mutant maize (Figure 6F). However, under drought stress, both the symbiotic and non-symbiotic *zmtip2;3* mutant maize exhibited significantly lower POD and SOD activities compared to the wild-type maize, with the symbiotic plants showing a highly significant difference (Figure 6G). This further suggested that *ZmTIP2;3* could enhance the activity of the POD and SOD enzymes in plants, thereby improving their ability to withstand drought stress. Furthermore, the ability for drought resistance was further enhanced under the induction of AM fungi.

To investigate the changes in intracellular toxic substances in symbiotic and non-symbiotic *zmtip2;3* mutant plants under drought stress, the content of H_2_O_2_ was measured. Under normal water conditions, there was no significant difference in H_2_O_2_ content between the *zmtip2;3* mutant and wild-type maize plants, whether inoculated with AM fungi or not (Figure 6H). However, after drought stress, both the symbiotic and non-symbiotic *zmtip2;3* mutant maize plants exhibited a significant increase in H_2_O_2_ content compared to the wild-type plants. Specifically, the content of H_2_O_2_ in the non-symbiotic and symbiotic *zmtip2;3* mutant plants increased by 19.4% and 20.2%, respectively, compared to the wild type. Moreover, the H_2_O_2_ content in the symbiotic *zmtip2;3* mutant and wild-type plants was lower than that in the non-symbiotic plants. These results indicated that symbiosis with AM fungi reduced the accumulation of H_2_O_2_ in plants and enhanced the drought resistance of maize. 

To further investigate the impact of AM fungi on cellular osmolytes under drought stress, the content of proline was measured. Under normal water conditions, there was no significant difference in the proline content between the non-inoculated wild-type and *zmtip2;3* mutant maize plants (Figure 6I). However, in the *zmtip2;3* mutant maize inoculated with AM fungi, the proline content decreased significantly by 28.9% compared to the wild type. Under water stress conditions, both the non-symbiotic and symbiotic *zmtip2;3* mutant plants showed a significant decrease in proline content compared to the wild-type plants, with a highly significant difference observed in the symbiotic plants, which were reduced by 40.1%. The results suggested that *ZmTIP2;3* influenced the proline content to adapt to drought stress, and AM fungi could induce *ZmTIP2;3* to enhance the plant’s drought resistance.

During periods of water deficiency, LEA proteins are crucial for maintaining other proteins, the vesicle or inner membrane structure, chelating ions (such as calcium ions), binding or substituting water, and acting as molecular chaperones during cellular dehydration [23,24]. *P5CS* is a key enzyme involved in plant proline synthesis, and its expression is increased in response to stress; the *NECD* gene, which encodes 9-homeopathic-epoxy carotenoid dioxygenase, is involved in the biosynthesis of the hormone ABA. In this study, we determined the changes in *LEA3*, *P5CS4*, and *NECD1* expression in the roots of wild-type and *zmtip2;3* mutant maize after inoculation with AM fungi for 8 weeks under normal water and drought treatment. The results indicated that there was no significant difference in the expression of *LEA3* and *P5CS4* between the wild-type and *zmtip2;3* mutant maize when well-watered without AM fungi symbiosis. The expression of *LEA3* in the *zmtip2;3* mutant roots uninoculated and inoculated with AM fungi was reduced by 32.1% and 40.1%, respectively, in contrast to the wild type under drought treatment (Figure 6J). The expression of *P5CS4* in the *zmtip2;3* mutant roots uninoculated and inoculated with AM fungi was reduced by 24.9% and 28.8%, respectively, compared to the wild type under drought condition (Figure 6K). The expression of *NECD1* in the *zmtip2;3* mutant roots uninoculated and inoculated with AM fungi was reduced by 27.1% and 46.6%, respectively, compared to the wild type under drought conditions (Figure 6L). 

## 3. Discussion

Plants developed their own defense systems to protect themselves from the impact of drought [25], for instance, by regulating the expression of aquaporin genes [5] and symbiosis with AM fungi [26] to enhance plant drought resistance. In this study, AM fungi induced the expression of the aquaporin gene *ZmTIP2;3* under drought conditions, thereby regulating maize physiological and biochemical reaction under drought stress and increasing maize drought resistance. This finding provides a feasible way to improve maize varieties to improve drought resistance and is expected to play an important role in agricultural production in the future.

In previous studies, AQPs have been identified in many other species, such as 41 in maize [27], 35 in *Arabidopsis* [28], 33 in rice [29], 41 in sorghum [30], and 66 in soybean [31]. In maize, PIP subfamily members have been reported more frequently in the past, while TIP subfamily members have been studied relatively less. Currently, the more extensively studied TIP genes include *ZmTIP1;1*, *ZmTIP1;2*, *ZmTIP2;1*, and *ZmTIP2;2*, which have been found to be associated with water transport and can improve the plant’s ability to withstand water stress. ZmTIP2;3 is an aquaporin belonging to the TIP subfamily, and is closely related to ZmTIP2;1 and ZmTIP2;2. Just as we speculated, *ZmTIP2;3* was induced by drought (Figure 3C). This is consistent with the study conducted by Zézé et al. (2008) [32], where they found that inoculating the roots of *Trifolium alexandrium* under drought stress with the Moroccan mycorrhizal vaccine *Aoufous Complex* could stimulate MIP expression levels. The preliminary transcriptome analysis and qPCR revealed that *ZmTIP2;3* is strongly induced by AM fungi (Figure 3A,C). Based on tissue expression patterns and quantitative data analysis, *ZmTIP2;3* exhibited higher expression levels in the roots compared to other tissues, so *ZmTIP2;3* was chosen for the subsequent functional analysis (Figure S1). Li et al. (2013) [33] found two genes, *GintAQPF1* and *GintAQPF2*, from *Rhizoglomus intraradices*, confirming that the AQP transports water through AMF under DS conditions. In drought conditions, AQPs may be involved in enhanced drought resistance through AMF, but this is unclear and controversial. Therefore, we aimed to investigate the role of *ZmTIP23* in maize drought resistance under AM fungi symbiosis conditions.

The plant biomass serves as a comprehensive indicator of plant growth under adversity. In this study, the results of the phenotypic analysis showed that the growth, leaf color, and root development of wild-type and *zmtip2;3* mutant maize inoculated with AM fungi were higher than those of the uninoculated wild-type and *zmtip2;3* mutant maize (Figure 4B). Meanwhile, the growth of the wild-type plants inoculated with AM fungi was also better than that of the *zmtip2;3* mutant inoculated with AM fungi. In the presence of AM fungi, the plant biomass was higher, and while the maize biomass decreased under drought stress, this reduction was partially compensated for by AMF symbiosis. Following the mutation of *ZmTIP2;3*, there was no significant difference in the biomass between the wild-type and *zmtip2;3* mutant plants under non-AM conditions, but the *zmtip2;3* mutant plants exhibited poorer growth under drought stress, indicating increased drought tolerance conferred by *ZmTIP2;3* (Figure 4C). This is consistent with the results of Nijjer et al. (2004) [34] in *Sapium sebiferum* and Özgönen et al. (2010) [35] in peanut. It is well known that AMF uses its widely dispersed mycelium to absorb water from the soil and deliver it to plant roots, absorbing nutrients and water for and greatly enhancing the growth ability of the host plant [36,37]. Therefore, an increase in plant development under mycorrhization could be the result of extraradical hyphae in AMF seedlings improving their ability to absorb water and nutrients. In general, an increase in AQP expression in plants under dehydration stress indicates an increase in the water permeability of the membrane, which facilitates the movement of water through the host plants [38]. The permeability of the membrane appears to be reduced if AQP expression is downregulated during dehydration stress, allowing the host cells to hold onto water [38]. According to our findings, DS considerably decreased the RWC in the AMF and non-AMF maize as compared to the WW (Figure 6E). Under well-watered conditions, there were no significant differences in the relative water content of the symbiotic and non-symbiotic *zmtip2;3* mutants compared to the wild type. These results indicate that the knockout of the *ZmTIP2;3* gene significantly reduced the ability for water absorption and transport under drought stress, leading to a decreased relative water content in the leaves, with a more pronounced effect under symbiotic conditions. These results indicated that the expression of *ZmTIP2;3* increased the water permeability of the membrane under drought stress, thus promoting water transport in the maize, resulting in a higher RWC in the wild-type maize than in the *zmtip2;3* mutant maize. On the other hand, AM fungi symbiosis induced the expression of the *ZmTIP2;3* gene, enhancing water absorption and transport, and thereby improving the drought resistance of the maize. The results of this study are in line with those of previous research on *Cupressus atlantica* and *Poncirus trifoliata* [37,39].

The results of the photosynthesis-related indexes, including the photosynthetic rate and stomatal conductance, showed that the photosynthetic-related indexes of the *zmtip2;3* mutant were lower than those of the wild type regardless of the moisture adequacy or drought stress condition, and the *zmtip2;3* mutant was subjected to more water stress compared with the wild type (Figure 6A–D). These results indicated that the *ZmTIP2;3* gene may be crucial for maize’s ability to transport water and resist drought stress. The activation of plant antioxidant systems by mycorrhizal fungi involves antioxidant enzymes such as catalase (CAT), superoxide dismutase (SOD), peroxidase (POD), glutathione peroxidase (GPX), and ascorbate peroxidase (APX) to balance the cell’s ROS levels and maintain REDOX homeostasis [40]. The present study’s findings clearly demonstrated how AMF inoculation protects plant cells by strengthening antioxidant enzyme systems. The antioxidant enzyme activity and proline content of the wild type and *zmtip2;3* mutant of the uninoculated AM fungi were higher than those of the uninoculated AM fungi under sufficient water and drought stress, and the biochemical indexes of the wild type were higher than those of the *zmtip2;3* mutant (Figure 6F–I). These results indicated that *ZmTIP2;3* promoted the drought resistance of maize plants, and was more effective in the wild type’s drought resistance after inoculation with AM fungi. Under drought stress, excess ROS can induce oxidative damage in plants by accelerating the metabolic accumulation of superoxides and hydroxyl radicals, hydrogen peroxide (H_2_O_2_), and monomer oxygen in vivo [41]. Most water stress tests have shown that mycorrhizal fungi can enhance oxidative stress in plants, reduce the accumulation of MDA and H_2_O_2_, and thus reduce the degree of oxidative damage to cells. H_2_O_2_ is thought to be an intermediary molecule involved in a number of physio-biochemical processes in plants in addition to being a toxic molecule that causes oxidative damage. In drought conditions, both the symbiotic and non-symbiotic *zmtip2;3* mutant maize plants exhibited a significant increase in H_2_O_2_ content compared to the wild-type plants (Figure 6H). Specifically, the content of H_2_O_2_ in the non-symbiotic and symbiotic *zmtip2;3* mutant plants increased by 19.4% and 20.2%, respectively, compared to the wild type. Moreover, the H_2_O_2_ content in the symbiotic *zmtip2;3* mutant and wild-type plants was lower than that in the non-symbiotic plants. These results suggested that symbiosis with AM fungi reduced the accumulation of H_2_O_2_ in the plants and protected the maize from oxidative stress, thereby enhancing the drought resistance of the host plant. The results obtained here are consistent with those reported in previous research on *Robinia pseudoacacia* L. [42].

The ability of AM fungi to enhance plant drought resistance is primarily achieved through the modulation of relevant genes. This regulation includes the modulation of gene expression within the AM fungi themselves or within host plants, thereby enhancing the plants’ ability to recover from stress brought by drought [43]. Drought stress-induced genes can be divided into two categories: functional genes and regulatory genes [44]. Functional genes refer to genes that play specific functions in acquiring drought resistance, such as aquaporin and transporter genes, genes related to osmotic regulation substances, late embryogenesis abundant (LEA) genes, and antioxidant-related enzyme genes, among others. Regulatory genes refer to genes that regulate the expression of functional genes, including certain plant hormone synthesis genes, transcription factors, and some regulatory non-coding RNAs. Currently, research in this area predominantly focuses on the potential regulation of genes encoding late embryogenesis abundant (LEA) proteins, aquaporins (AQPs), ∆1-pyrroline-5-carboxylate synthetase (P5CS), and 9-cis-epoxy carotenoid dioxygenase (NECD) during late embryo development. The latter is an essential enzyme in the process of ABA biosynthesis [23,45]. The LEA protein is a late embryogenesis abundant protein (LEA protein). When plants are subjected to stress, the LEA gene is induced to express in various nutritional tissues, thereby enhancing plant tolerance to stress [46,47]. A previous report found that rice’s ability to withstand drought was improved by overexpressing *OsEm1*, the gene that codes for a group I LEA protein, which raises ABA sensitivity and improves osmotic tolerance [48]. In our study, we found that the expression of *LEA3* in the *zmtip2;3* mutant roots uninoculated and inoculated with AM fungi was reduced by 32.1% and 40.1%, respectively, in contrast to the wild type under drought treatment (Figure 6J). Proline is an important regulatory substance for wheat resistance to drought stress, and Pyrrpline-5-carboxylate synthetase (*P5CS*) is a crucial enzyme for proline synthesis during drought conditions. Kobra et al. [49] found that under drought stress conditions, lines with strong drought resistance exhibited higher *TaP5CS* gene expression and proline accumulation than lines with weak drought resistance. The expression of *P5CS4* in the *zmtip2;3* mutant roots uninoculated and inoculated with AM fungi was reduced by 24.9% and 28.8%, respectively, compared to the wild type under drought conditions (Figure 6K). *ZmTIP2;3* improved the drought tolerance of the maize plants, so the proline content of the wild type was higher than that of the mutant type, and the expression of *P5CS* was correspondingly higher in the wild type than that of the mutant type. Inoculation with AM fungi can improve the drought resistance of maize plants, especially the wild type. The increase in the proline content reduces the damage to cells caused by stress. Therefore, the expression of *P5CS* in the wild type of mycorrhiza is higher than that of the *zmtip2;3* mutant of mycorrhiza. The expression of *NECD1* in the *zmtip2;3* mutant roots uninoculated and inoculated with AM fungi was reduced by 27.1% and 46.6%, respectively, compared to the wild type under drought conditions (Figure 6L). NECD is a critical enzyme in the ABA biosynthesis pathway. Under drought stress, the elevation of ABA levels represents a typical physiological response in plants to enhance drought resistance [50]. Therefore, under drought conditions, the expression of *NECD1* in the roots of the *zmtip2;3* mutants increased compared to that in well-watered conditions, and the expression of *NECD1* in the roots of the *zmtip2;3* mutants inoculated with AM fungi was higher than in those not inoculated (Figure 6L). However, different plants exhibit varying levels of response to ABA after inoculation with AM fungi. Under drought conditions, the ABA levels in mycorrhizal plants are not always higher than in non-mycorrhizal plants [51,52,53].

## 4. Materials and Methods

### 4.1. Plant Materials and Growing Environment Used in the Experiment

#### 4.1.1. Plant Materials

The study materials maize wild-type B73 inbred line and *Lotus japonicus* (*L. japonicus*) MG20 were from the Key Laboratory of Crop Stress Resistance and High Quality Biology of Anhui Province of Anhui Agricultural University, and the AM fungi species was *Glomus intraradices* (provided by Sun Yat-Sen University, Guangzhou, China). The AMF inoculum comprised dry sand, mycelium, spores, and root fragments, cultivated on maize for four months. Approximately five spores are present per gram of inoculum.

The *zmtip2;3* mutant of B73 background maize was screened from the maize EMS mutant library of Professor Lu Xiaoduo from Anhui Agricultural University. Utilizing the EcMutMap (exome capture mutation mapping)-assisted positioning technique [54], the relevant gene Zm00001d026177 (*ZmTIP2;3*) was obtained, wherein a C/T mutation occurred at the 376th nucleotide of the CDS, resulting in premature termination of translation. Subsequent to seeding, genomic DNA was extracted from leaves, and amplification was conducted using the forward primer 5′-TTCTACTGCCTCCTCTCATACC-3′ and the reverse primer 5′-ATCACGATCTCCATCACCAC-3′ to validate the presence of the mutated gene through sequencing. Thereafter, the mutant was backcrossed with B73 as the recurrent parent, and the mutation was screened using the aforementioned method. Through successive backcrossing of mutant plants, followed by four generations of backcrossing, homozygous mutants were obtained via self-crossing for subsequent gene functional analysis.

#### 4.1.2. Plant Growth and AMF Inoculation

Maize seeds were surface-disinfected with 6% bleach for 10 min and rinsed three times with deionized water. Following washing, the seeds were allowed to sprout for seven days at 28 °C on seed germination paper with a 16 h light/8 h dark cycle. Following the methodology described by Quiroga et al. in 2019 [55], improvements were made to the cultivation and treatment of maize, involving two water treatments (sufficient water and drought) and two mycorrhizal inoculation treatments (non-inoculation and *G. intraradices* spore inoculation (200 spores per plant)). The wild type (B73) and zmtip2;3 mutant seedlings were transplanted into pots containing a vermiculite, sand, and perlite (6:1:1) mixture. The pots were then cultivated in a greenhouse with a 16 h light/8 h dark cycle at 25 °C/20 °C. Soil moisture was kept at 70–75% of field capacity for the first 6 weeks after sowing, with weekly applications of Hoagland solution until harvest. Subsequently, half of the maize plants inoculated with AMF and half of the non-inoculated maize plants were subjected to drought treatment until soil moisture reached 40–45% of field capacity. The remaining half of the plants were maintained under normal field moisture conditions and grown for 14 days. Soil moisture was monitored using a soil moisture meter (Fieldscout TDR350, Spectrum Technologies, South Industrial Drive East, Plainfield, IL, USA).

### 4.2. RNA Isolation and qRT-PCR

The phenol–chloroform extraction procedure was utilized to isolate maize total RNA, and the HiScript II 1st Strand cDNA Synthesis Kit (Vazyme, Nanjing, China) was utilized to synthesize cDNA. Using ChamQ Universal SYBR qPCR Master Mix (Vazyme, Nanjing, China), qRT-PCR was carried out on an PikoReal Real Time PCR System (TCR0096, Thermo Scientific, Waltham, MA, USA) in accordance with the manufacturer’s instructions. As internal controls for maize, the expression of reference genes *Zmα-Tubulin* and *ZmGAPDH* was utilized. Three biological replicates per sample were used to compute the relative gene expression using the 10^–(ΔCt/3)^ method after the expression levels were normalized to those of non-inoculated AMF maize [56]. Table S1 contains a list of the primers used in qRT-PCR.

### 4.3. Bioinformatics Analysis

The neighbor-joining method was used to create the phylogenetic tree using MEGA7 software (version 11.0.10). Bootstrap testing was conducted with 1000 iterations, and the model used was the Poisson model. The phylogenetic tree was beautified using the online visualization tool chiplot (https://www.chiplot.online/tvbot.html, accessed on 2 January 2024) [57]. Multiple sequence alignment of aquaporin sequences, including ZmTIP2;3, was performed using CLUSTALW (https://www.genome.jp/tools-bin/clustalw, accessed on 2 January 2024), and the aligned protein sequences were then submitted to GENEDOC software (version 2.7) to obtain a protein homology map. Known aquaporin reference numbers in NCBI include AtTIP2;2 (NP_193465.1), OsTIP1;1 (NP_001388972.1), HvTIP1;1 (XP_044982938.1), PtTIP1;2 (XP_01564586), PgTIP1 (ABB29477), ZmPIP2;3 (NP_001105025). The TMHMM website (http://www.cbs.dtu.dk/services/TMHMM/, accessed on 2 January 2024) was used to predict the transmembrane structural domain. The RSAT website (https://rsat.eead.csic.es/plants/dna-pattern_form.cgi, accessed on 2 January 2024) was utilized for the analysis and visualization of the cis-acting elements present in the *ZmTIP2;3* promoter. The heatmap was generated using the TBtools software (version 2.042).

### 4.4. Vector Construction

To create p*ZmTIP2;3*::GUS, we amplified a fragment of 1786 bp upstream of the *ZmTIP2;3* ATG start codon and inserted it into the pCAMBIA1301 vector. *Nco* I and *Hind* III restriction enzyme sites were included in the primers. For constructing the subcellular localization vector *ZmTIP2;3*::GFP, under the control of the CaMV35S promoter, the coding sequence of *ZmTIP2;3* was amplified using the ClonExpress II One Step Cloning Kit (Vazyme, Nanjing, China) and cloned into the pCAMBIA1305 vector. *Spe* I and *BamH* I restriction enzyme sites were included in the primers. Table S2 lists the primers used for vector construction.

### 4.5. Subcellular Localization in Maize Protoplasts

The *ZmTIP2;3*::GFP and 1305::GFP plasmids were cotransformed with a nucleus or a plasma membrane marker (mcherry) plasmid into maize protoplast using PEG. After 16 h, the fluorescent signals of mcherry and green fluorescent protein (GFP) were detected and photographed under a confocal microscope (Leica LSM800, Weztlar, Germany) with excitation wavelengths at 514 nm and 488 nm. The empty 1305::GFP was used as a control.

### 4.6. Induced Transformation of Hairy Roots of L. japonicus 

*L. japonicus* seeds were rubbed with sandpaper, washed with 75% ethanol for 30 s, washed with 12% bleach solution plus 2 drops of Tween for 10 min, washed with 75% ethanol three times (defoaming), each time for 1–2 min, and washed with sterile water 3–5 times, each time for 4–5 min. The sterilized seeds were placed in a refrigerator for 12 h at 4 °C for vernalization treatment, and then the seeds were spread on 1–1.2% water agar culture plates on a clean bench, sealed with sealing film to prevent contamination by miscellaneous bacteria, and incubated in the dark in the incubator, first upright for 24 h, then inverted for 24 h, until the roots of the germinated seeds grew to about 1 cm.

The correct recombinant vector p*ZmTIP2;3*-GUS was electroporated into the *Agrobacterium tumefaciens* strain LBA9402, spread on YEB solid medium, and verified by selecting single colonies grown on the medium. The positively verified colonies on the YEB solid medium plates were then spread onto YMB solid medium and cultured in the dark at 28 °C for 48 h to grow extensive colonies. Sterile B&D (Broughton and Dilworth) medium was supplemented with 150 µL acetylsyringone, poured into square culture plates placed at a slight incline. From the thinner side of the solidified B&D medium, about 1/3 of the size was cut off, and incisions about 1 cm long were made in the remaining medium. For two days, root embryos of *L. japonicus* were excised from the root tip to the root hair region using a sterile surgical blade, infected with *Agrobacterium tumefaciens* strain LBA9402 transformed with p*ZmTIP2;3*-GUS, and then transferred to the incisions on the B&D medium. The culture plates were sealed with sealing film and medical adhesive tape to prevent contamination by miscellaneous bacteria. The B&D culture plates were initially cultured in a 28 °C incubator in the dark for 24 h, and then in a 23 °C incubator in 16 h light/8 h darkness, and grown for approximately 3 weeks.

We selected hairy roots of *L. japonicus* with swollen and branching root hairs grown for approximately 3 weeks on B&D medium for transplantation. The planting substrate consisted of a mixture of vermiculite, perlite, and AM fungi (*G. intraradices*). We cultivated the roots in a greenhouse at 25 °C for 8 weeks under a photoperiod of 16 h light and 8 h darkness. After cultivation, we washed the harvested hairy roots of *L. japonicus*, stained the roots with GUS, and incubated them in the dark at 37 °C for 24 h. If blue staining appeared on the roots, we prepared slides and observed under a microscope for photography.

### 4.7. Detection of Mycorrhizal Infection Rate 

First, the roots of maize and *L. japonicus* were fixed in FAA solution for more than 4 h. Then, the roots were removed and transparentized in 10% KOH solution, then heated for an hour at 90 °C. Subsequently, the cooled roots were removed from the 10% KOH, washed, and treated with an appropriate amount of 5% lactic acid, followed by a 30 min immersion in a 90 °C water bath. Finally, the roots were treated with a lactic acid and glycerol solution on a shaker to make them translucent after being stained for 24 h with a 0.05% trypan blue solution. Fifty 1 cm long root segments were placed on slides and examined for particular AMF structures using a Leica DM5000B microscope (Leica, Weztlar, Germany).

The method described by Trouvelot et al. (1986) [58] was used to classify the AMF colonization rate of plant root segments into six levels: 0, <1%, <10%, <50%, <70%, and >90%. The parameters F%, M%, and m% were calculated using Excel. The biological significance of each parameter and their calculation formulas are as follows:

F% (frequency of mycorrhiza in the root system, infection rate) reflects the degree of AMF colonization in the root segments of host plants and is calculated as follows: F% = (the number of roots infected by AMF in the sample/the total number of roots in the sample) × 100.

M% (intensity of the mycorrhizal colonization in the root system) is a weighted average that comprehensively reflects the AMF colonization rate and colonization intensity in all root segments. The calculation method for M% is M% = (95 × n5 + 70 × n4 + 30 × n3 + 5 × n2 + n1)/(total number of root segments of the sample); (n5) represents the number of root segments with AMF infection intensity more than 90%, (n4) represents the number of root segments with AMF infection intensity of 70%, (n3) represents the number of root segments with AMF infection intensity of 50%, (n2) represents the number of root segments with AMF infection intensity of 10%, and (n1) represents the number of root segments with AMF infection intensity of 1%.

m% (intensity of the mycorrhizal colonization in the root fragments) reflects the formation intensity of AMF in root segments. The calculation method for m% is m% = M × (total number of root segments)/(number of infected root segments), which comprehensively reflects the frequency and intensity of AMF infection in all infected root segments.

### 4.8. Determination of Physiological and Biochemical Indicators

The photosynthetic indices of the second fully expanded leaves at the top of the maize plants were measured on sunny days between 10:00 a.m. and 1:00 p.m. using a photosynthetic instrument (LI-6400, LI-COR, USA). After 8 weeks of symbiosis with AMF, maize leaves and roots were harvested under both well-watered and drought conditions, and their fresh and dry weights were determined. Fresh weight refers to the weight of harvested leaves or roots, while dry weight refers to the weight of leaves or roots after drying to constant weight in an oven. Eight weeks after symbiosis with AMF, maize leaves were harvested under well-watered and drought conditions, and physiological and biochemical parameters were measured. The expression levels of drought-related genes *LEA3*, *P5CS4*, and *NECD1* were determined. The POD activity, SOD activity, H_2_O_2_ content, and proline content were determined using assay kits (BC0095 for POD, BC0175 for SOD, BC3595 for H_2_O_2_, and BC0295 for proline, Solarbio, Beijing, China). POD, H_2_O_2_, and proline were measured using a UV spectrophotometer, while SOD was measured using a microplate reader.

### 4.9. Determination of Relative Water Content of Leaves

We harvested fully expanded maize leaves and measured their fresh weight (FW). Next, we immersed the leaves in distilled water until saturated, dried them with filter paper, and weighed them (TW). Finally, we dried the leaves in an oven to a constant weight and measured the dry weight (DW). The calculation formula for leaf relative water content (RWC) is RWC (%) = (FW − DW)/(TW − DW) × 100%.

### 4.10. Statistical Analysis

The data were obtained from three independent replicate experiments, processed using Excel 2019, and plotted using GraphPad Prism (version 8.0.2). Student’s *t-*test was employed to identify significant differences, where * *p* ≤ 0.05 indicates significant difference, ** *p* ≤ 0.01 indicates highly significant difference. “ns” represents no significant difference.

## 5. Conclusions

ZmTIP2;3 is a membrane-localized aquaporin with six transmembrane domains and two highly conserved NPA motifs. Its promoter region contains many cis-acting elements associated with mycorrhiza induction. *ZmTIP2;3* was highly induced under drought conditions through symbiosis with AM fungi. The mutant *zmtip2;3* displayed reduced biomass, colonization rate, photosynthesis, POD and SOD activity, proline content, and expression levels of several drought-related genes (*LEA3*, *P5CS4*, and *NECD1*) in comparison to the wild type after being inoculated with AM fungi under drought stress. This suggested that *ZmTIP2;3* improved the drought resistance in maize through symbiosis with AM fungi.

## Data Availability

The data presented in this study are available in the article or Supplementary Materials.

## Supplementary Materials

The following supporting information can be downloaded at https://www.mdpi.com/article/10.3390/ijms25084205/s1.
