# Supplementary material for: Aquaporin ZmTIP2;3 Promotes Drought Resistance of Maize through Symbiosis with Arbuscular Mycorrhizal Fungi
Source: Int J Mol Sci. 2024 Apr 10;25(8):4205. doi: 10.3390/ijms25084205 (PMC11050007; doi:10.3390/ijms25084205)
Supplement: Supplementary file 1 [file ijms-25-04205-s001.zip › ijms-2921477-supplementary.pdf]

Supplementary data.

Table S1 Primers for qRT-PCR.

Primer name	Primer Sequence	Primer name	Primer Sequence
ZmTIP2;3-qPCR-F	TCGCATTTGGAAGCTTTCGC	*ZmTIP2;3*-qPCR -R	CTTCGTCAATTGCGAGTAGGC
Zmα-Tubulin-F	TCCTGGACAACGAGGCTATCTAT	*Zmα-Tubulin*-R	TGTGAGATCAGCCTGTTCAAGTT
ZmGAPDH-F	AGCAGGTCGAGCATCTTCG	*ZmGAPDH*-R	CTGTAGCCCCACTCGTTGTC

Table S2 Primers for vector construction.

Primer name	Primer Sequence	Primer name	Primer Sequence
ZmTIP2;3-F	ATGGTGAAGCTCGCATTTGG	*ZmTIP2;3*-R	CGAGTAGGCAATGGCGGA
ZmTIP2;3-GFP-F	ggacagcccagatcaactagtATGGTGAAGCTCGCATTTGG	*ZmTIP2;3*-GFP-R	gcccttgctcaccatggatccTGGGTACTCCTGCTGGCCG
p*ZmTIP2;3*-F	CTACAACCGTACAGGCAAAAAGT	p*ZmTIP2;3*-R	CTTTTTTCCCCCCCTCTCC
p*ZmTIP2;3*-GUS-F	gacctgcaggcatgcaagcttCTACAACCGTACAGGCAAAAAGTATT	p*ZmTIP2;3*-GUS-R	ttaccctcagatctaccatggCTTTTTTCCCCCCCTCTCC

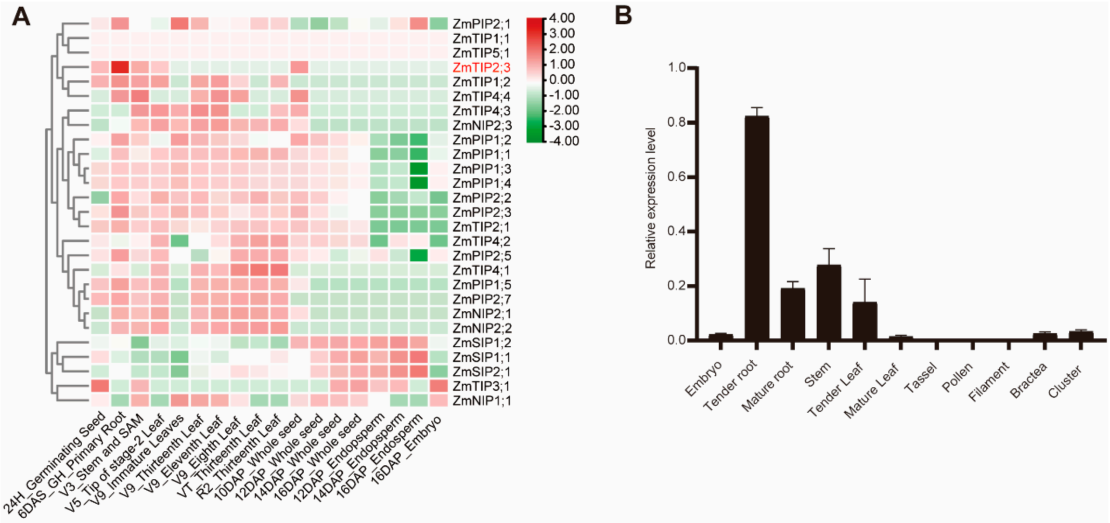

Figure S1. Tissue expression pattern analysis of *ZmTIP2;3*. (A) Heatmap of the tissue expression pattern of *ZmTIP2;3*. (B) The relative expression level of *ZmTIP2;3* in different tissues of maize was measured by qRT-PCR.
